# Syndrome of duodenal compression by the superior mesenteric artery following restorative proctocolectomy: a case report and review of literature

**DOI:** 10.1590/S1516-31802005000300013

**Published:** 2005-05-02

**Authors:** Jaques Waisberg, Maria Helena de Toledo Zewer, Antonio Claudio de Godoy

**Keywords:** Superior mesenteric artery syndrome, Duodenal obstruction, Superior mesenteric artery, Restorative proctocolectomy, Síndrome da artéria mesentérica superior, Obstrução duodenal, Artéria mesentérica superior, Proctocolectomia restauradora

## Abstract

**CONTEXT:**

Restorative proctocolectomy with anastomosis of an ileal pouch to the anal canal is a new and rare cause for triggering the syndrome of duodenal compression by the superior mesenteric artery. Restorative proctocolectomy requires assessment of the position of the duodenum in relation to aortomesenteric constriction to avoid the occurrence of duodenal compression by the superior mesenteric artery.

**CASE REPORT:**

The authors report on a case of this syndrome in a patient with familial adenomatous polyposis and review the literature on the etiopathogenesis, diagnosis, treatment and prevention of this unusual entity.

## RESUMO

**CONTEXTO:**

A proctocolectomia restaurativa com anastomose de bolsa ileal no canal anal é causa nova de desencadeamento da síndrome da compressão duodenal pela artéria mesentérica superior. A proctocolectomia restaurativa requer avaliação da posição do duodeno em relação ao pinçamento aortomesentérico para evitar a sua compressão pela artéria mesentérica superior.

**RELATO DE CASO:**

Os autores relatam um caso desta síndrome em doente com polipose adenomatosa familiar e realizam revisão da literatura no tocante à etiopatogênese, diagnóstico, tratamento e prevenção desta inusitada entidade.

## INTRODUCTION

The syndrome of duodenal compression by the superior mesenteric artery is directly related to an anatomical and mechanical factor and to acute or chronic reduction of the retroperitoneal fat.^1^

Several debilitating pathological conditions that have in common marked weight loss (multiple trauma, sepsis, anorexia nervosa and burns) diminish the retroperitoneal fatty padding and can trigger the syndrome.^1^ In such situations and as a result of man's supine resting position, in contrast to the position of four-legged mammals, the angle formed by the aorta and the superior mesenteric artery changes from 90° to an accentuated acute angle, thus forming vascular constriction at the location where the duodenum usually crosses and thereby triggering the syndrome.^1^

Restorative proctocolectomy with anastomosis of an ileal pouch to the anal canal has become the surgery of choice for young patients with ulcerative colitis and patients with familial adenomatous polyposis. This surgical procedure has become a new and rare cause of the syndrome of duodenal compression by the superior mesenteric artery, with only seven cases published to date^2-7^ (Table 1).

**Table 1:** Superior mesenteric artery syndrome after restorative proctocolectomy: cases in the literature

Author/date	Disease	Diagnostic method	Treatment		Outcome
			Clinical	Surgical	
Ballantyne et al.^2^ 1987	URC	CT scan	NGP 14 days	No	Good
Christie et al.^3^ 1988	URC	X-ray contrast	NGP + PPN 42 days	Duodenal division and re-anastomosis anteriorly to the SMA	Good
Tonelli et al. 1993^4^	1^st^ - FAP	X-ray contrast	NGP + PPN 18 days	No	Good
	2^nd^ - URC	CT scan	NGP + PPN 14 days	No	Good
Goes et al.^5^ 1995	URC	X-ray contrast	NGP 7 days	Strong's operation	Good
Ravindra et al.^6^ 1999	URC	X-ray contrast	NGP 7 days	Duodenal division and re-anastomosis anteriorly to the SMA	Good
Essadel et al.^7^ 2001	URC	X-ray contrast	NGP 7 days	Gastrojejunal anastomosis	Good
Present case	FAP	CT scan	NGP + PPN 17 days	No	Good

*NGP = nasogastric tube; PPN = prolonged parenteral nutrition; URC = ulcerative rectocolitis; FAP = familial adenomatous polyposis; SMA = superior mesenteric artery.*

The objectives of the present study were to describe a new case of syndrome of duodenal compression by the superior mesenteric artery after restorative proctocolectomy and to review the literature concerning the etiopathogenesis, diagnosis, treatment and prevention of this rare syndrome.

## CASE REPORT

A 20-year-old white woman, of weight 45 kg and height 1.68 meters (body mass index = 15.9 kg/m^2^), with familial adenomatous polyposis, was submitted to restorative proctocolectomy with manual anastomosis of a J-shaped ileal pouch to the anal canal and ileostomy in a protection loop. The mesentery was totally freed as far as its root and the ileocolic artery was ligated close to where it emerges from the superior mesenteric artery. The anastomosis performed was considered to be free from tension. Anatomopathological examination of the surgical specimen confirmed the diagnosis.

On postoperative day three, the nasogastric tube was removed and an oral diet was initiated. The patient presented two episodes of vomiting that intensified on the following day, accompanied by pain and epigastric fullness. The ileostomy continued to function adequately. A nasogastric tube was introduced again, with drainage of abundant gastric and bilious secretions.

**Figure 1:**
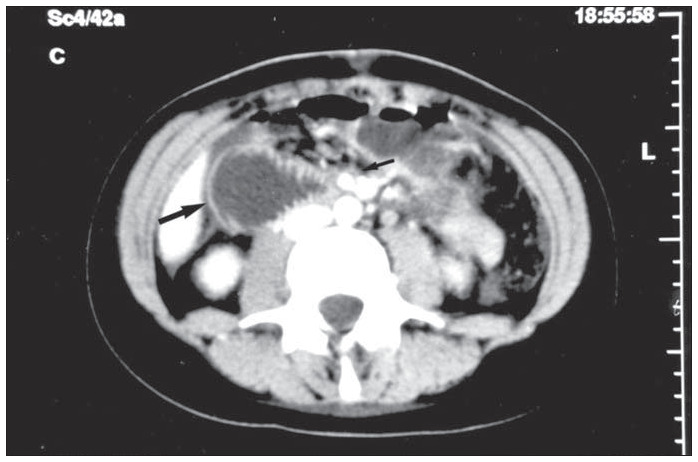
Abdominal computed tomography in the early postoperative period showing duodenal distension with signs of forced contraction (large arrow) and abrupt obstruction at the level of the aortomesenteric constriction (small arrow).

Computed tomography scanning of the abdomen revealed large-scale gastric and duodenal distension with stasis. There were signs of forced contractions in the duodenum and abrupt obstruction at the level of the aortomesenteric constriction, as well as a marked reduction in the retroperitoneal and perivascular fat (Figure 1). The signs thus confirmed the diagnosis of the syndrome of duodenal compression by the superior mesenteric artery.

**Figure 2:**
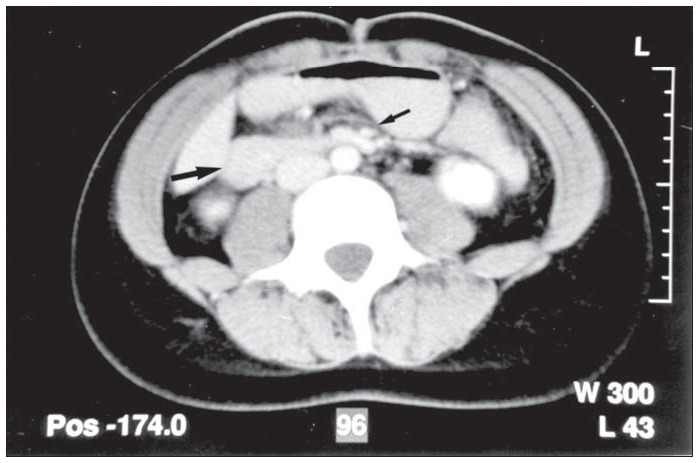
Abdominal computed tomography after prolonged hypercaloric parenteral nutrition showing absence of duodenal distension (large arrow) and replacement of the retroperitoneal adipose stocks at the level of the aortomesenteric constriction (small arrow).

Prolonged parenteral nutrition was instituted, which lasted for 17 days and probably replaced the retroperitoneal adipose stocks (Figure 2). After this, it was possible to remove the gastric tube and progressively reintroduce oral feeding to the patient. She was discharged on postoperative day 29 in a good condition, weighing 56 kg (body mass index =19.5 kg/m^2^).

## DISCUSSION

Anastomosis of an ileal pouch in the anal canal creates conditions for the accentuation of the angle between the aorta and the superior mesenteric artery.^1,3,4^ In the current case, albeit consisting of familial adenomatous polyposis disease, the patient presented weight loss, which may have also contributed to the appearance of the syndrome.

Radiological contrast examination and/ or computed tomography scan are considered to be appropriate for the diagnosis.^1,3,7^ Computed tomography scan presents the advantage of providing an overall assessment of the abdominal cavity and clearly demonstrating the location of the duodenal obstruction between the aorta and the superior mesenteric artery,^1,2,3,6^ as observed in the present report. It also contributes towards the diagnosis of other complications that may be acting to maintain the prolonged ileum or intestinal obstruction.

The initial treatment of this syndrome is clinical.^2,3,5,6^ Decompression via a nasogastric tube and prolonged hypercaloric parenteral nutrition to attempt to replace the retroperitoneal adipose stocks was shown to be effective in three of the cases described,^2,4,5^ and also in the present case, with an average prolonged parenteral nutrition duration of 16.3 days. Prolonged hypercaloric parenteral nutrition may be used before the restorative proctocolectomy to avoid the presentation of syndrome of duodenal compression by the superior mesenteric artery.

Strong^1^ was the first to successfully utilize the operation that now bears his name, for treating a case of syndrome of duodenal compression by the superior mesenteric artery. This author proposed the sectioning of the peritoneum anteriorly and superiorly between the duodenum and the pancreas, and posteriorly and inferiorly between the duodenum and the posterior peritoneum, as well as sectioning Treitz's ligament. In this way, the duodenum was freed to a position more distant from the apex of the angle between the two arteries. This decision was based on the anatomical and mechanical etiology since, according to Strong,^1^ the syndrome occurs because the duodenum is positioned and immobilized within this vascular pinching at its most acute position, because of its peritoneal attachments and the traction exerted by Treitz's ligament in the cranial direction. It is proposed that in patients with scant mesentery fat and a small-bowel mesentery under tension from ileal-anal anastomosis, consideration should be given to using Strong's technique for mobilizing the distal duodenum and thereby avoiding the presentation of syndrome of duodenal compression by the superior mesenteric artery in the postoperative period.^1 2 3^

The initial treatment is clinical and, whenever necessary, Strong's operation appears to be the best option in the surgical treatment of this syndrome, thus avoiding the risks inherent to anastomosis.^3,5-7^ With knowledge of the syndrome of duodenal compression by the superior mesenteric artery, its presence during the immediate postoperative period following restorative proctocolectomy may be suspected when physician faced with an extended ileum and suspected early obstruction. The utilization of computed tomography scanning is then recommended.^1-3^ This may increase the number of cases of the syndrome of duodenal compression by the superior mesenteric artery that are diagnosed and thus, in the future, may provide a firm basis for justifying the adoption of prophylactic surgical measures.
